# A44 STAT1 ACTS IN INNATE IMMUNE CELLS TO PREVENT LIVER PATHOLOGY FOLLOWING ASYMPTOMATIC INTESTINAL VIRAL INFECTION

**DOI:** 10.1093/jcag/gwad061.044

**Published:** 2024-02-14

**Authors:** A J Sharon, M Portas, B Hardman, L Osborne

**Affiliations:** Microbiology and Immunology, University of British Columbia, Vancouver, BC, Canada; Microbiology and Immunology, University of British Columbia, Vancouver, BC, Canada; Microbiology and Immunology, University of British Columbia, Vancouver, BC, Canada; Microbiology and Immunology, University of British Columbia, Vancouver, BC, Canada

## Abstract

**Background:**

In the mammalian gut, interactions between the host and commensal organisms must be regulated to prevent damage and derive benefits from these organisms. While this regulation has been well-studied in the context of bacteria, much less is known about how commensal-like viruses in the intestine are managed by the host.

Evidence demonstrates that viral commensals can provide benefits to the host which supplement those from bacteria. In mice, depletion of the intestinal virome enhances susceptibility to DSS-induced colitis. Contributions of the virome can also be studied using murine norovirus strain CR6 (CR6), which forms persistent asymptomatic infections in the gut; this infection protects mice from both *C. rodentium*- and DSS-induced colitis. Despite these benefits, host-encoded mechanisms regulating viral commensalism remain understudied.

One host-encoded mechanism is STAT1. In contrast to asymptomatic infection in wild-type mice, STAT1-deficient mice (*Stat1*^-/-^) develop severe disease characterized by liver necrosis and weight loss following CR6 infection. However, it remains unknown how the loss of STAT1 leads to CR6-induced disease.

**Aims:**

We aimed to define STAT1-dependent mechanisms of protection from CR6-induced liver pathology.

**Methods:**

To determine the contribution of STAT1 in protection from CR6-induced disease, *Stat1*^-/-^ and STAT1-sufficient *Stat1*^+/-^ littermates were infected i.v. with CR6. At days 3, 5, and 7 days post-infection, clinical disease, cell-intrinsic viral loads, pathology, and cellular infiltration were assessed. Bone marrow chimeras were used to evaluate the requirement for hematopoietic vs non-hematopoietic STAT1 in preventing disease.

**Results:**

By 3 days post-infection, myeloid cells infiltrate the liver of CR6-infected Stat1^-/-^ mice. Clinical disease is associated with accumulation of myeloid cells, including macrophage and dendritic cells in the liver . Sorting of liver-infiltrating immune cell populations revealed that *Stat1*^*-/-*^ macrophage have elevated viral loads compared to *Stat1*^+/-^ littermates (Figure). Notably, CD11b+ cells were enriched within the necrotic lesions characteristic of CR6-induced disease. Consistent with a role for innate immune cells in disease, bone marrow-derived macrophage from *Stat1*^-/-^ mice failed to control viral replication *in vitro*. Further, bone marrow chimeras revealed that STAT1 expression in hematopoietic cells is necessary and sufficient to prevent CR6-induced disease. Together, these data suggest that in the absence of STAT1, innate immune cells become heavily infected with CR6 and mediate severe liver pathology.

**Conclusions:**

Our data suggest that STAT1 is critical to maintaining a commensal relationship with the intestinal virome. In the absence of STAT1, failure to restrict the replication of viral commensal CR6 in innate immune cells leads to severe liver pathology.

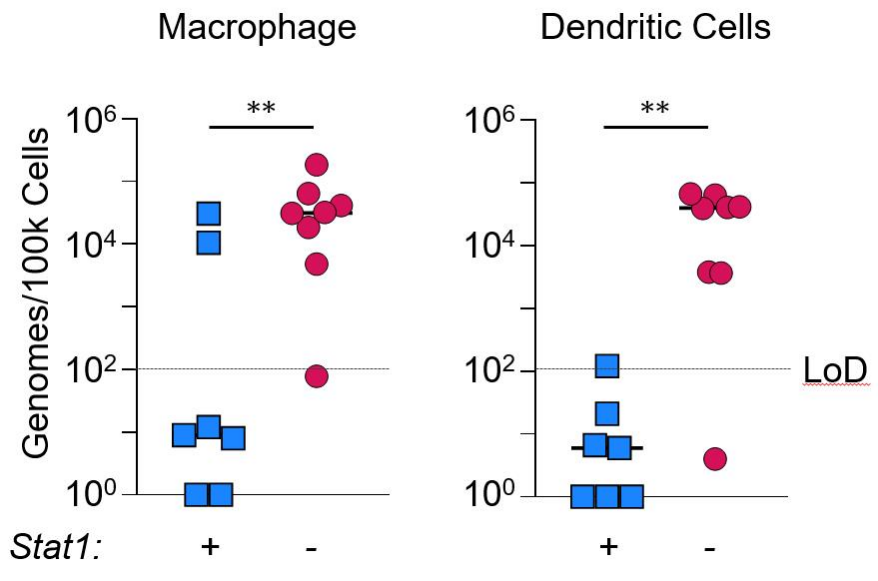

**STAT1-deficient innate immune cells fail to control viral replication.** *Stat1* ^*-/-*^ and *Stat1*^*+/-*^ mice were infected i.v. with CR6. 5 days post-infection, macrophage, dendritic cell, neutrophil, and monocyte populations were isolated from the liver by FACS. Viral genome copies were quantified by RT-qPCR. LoD = Limit of Detection. Mann-Whitney U test, ** = p ampersand:003C 0.01.

**Funding Agencies:**

CIHR

